# Allergen immunotherapy in Italy: How, when, and why—A real-world study conducted through a patient association

**DOI:** 10.1016/j.waojou.2024.101015

**Published:** 2024-12-24

**Authors:** Giovanni Paoletti, Emanuele Nappi, Maria Chiara Bragato, Paola Valli, Mattia Giovannini, Domenico Gargano, Luca Pecoraro, Deborah Diso, Simona Barbaglia, Giorgio Walter Canonica, Enrico Heffler

**Affiliations:** a Department of Biomedical Sciences, Humanitas University, Pieve Emanuele, MI, Italy; b Personalized Medicine, Asthma and Allergy, IRCCS Humanitas Research Hospital, Rozzano, MI, Italy; c Allergy Unit, Meyer Children's Hospital IRCCS, Florence, Italy; d Department of Health Sciences, University of Florence, Florence, Italy; e Allergy Unit, Azienda Ospedaliera S. Giuseppe Moscati, Avellino, Italy; f Pediatric Unit, Department of Surgical Sciences, Dentistry, Gynecology and Pediatrics, University of Verona, Verona, Italy; g Associazione Nazionale Pazienti Respiriamo Insieme-APS, Padua, Italy

**Keywords:** Allergen immunotherapy, Allergic rhinitis, Asthma, Costs, Effectiveness

## Abstract

**Background:**

Allergen immunotherapy (AIT) is the only treatment that modifies the natural course of allergies. However, AIT is only used in some eligible patients, is frequently underused, and only a few studies investigated this aspects. Understanding AIT utilization patterns might disclose information about why it is underused, thus providing valuable insights on how to broaden the positive impact it can have on the population.

**Methods:**

A questionnaire aiming to assess the trends of the use of AIT in Italy, patient health literacy on AIT, and the impact of respiratory allergies and AIT on patients' lives was submitted to patients affiliated with Respiriamo Insieme APS patient's association during the period between May and October 2023.

**Results:**

Nine hundred forty-four patients completed the questionnaire. Most patients reported to be affected by allergic rhinitis (81.1%), allergic asthma (45.4%), and/or allergic conjunctivitis (41.2%), and many of them presenting a combination of these diseases. Only 53.8% knew about AIT's existence, and AIT was proposed to only 33.1% of the 858 patients affected by allergic rhinitis and/or allergic asthma, of which 29.2% decided not to initiate the therapy. Common reasons for AIT refusal were related to indecisiveness (26.5%), costs (22.9%), and skepticism (19.3%). Among the remaining 70.8% who accepted to start AIT, 21.4% discontinued the treatment beforehand, on average 18.26 months from initiation. There was a high heterogeneity in the suggested duration of AIT, with 20.4% of patients receiving indications to continue AIT for less than 3 years. AIT positively impacted patients' lives as most patients who completed AIT found it effective and safe, and experienced a significant reduction in the workdays/schooldays lost due to disease exacerbations.

**Conclusions:**

The outcomes of this research pointed out a lot of misinformation surrounding AIT, calling for improvements in awareness and information on its efficacy and safety. Also, we noted a significant reduction in work/school days lost in patients who completed AIT and a good patient-reported safety and efficacy profile. We advocate for better economic accessibility through national-level standardization in treatment refundability. Notably, the collaboration with the patient association was crucial, and it would have been challenging to conduct this research otherwise.

## Introduction

Respiratory allergies contribute significantly to morbidity worldwide and typically affect the upper respiratory tract (allergic rhinitis - AR), the eyes (allergic conjunctivitis - AC), and/or the bronchial pathways (allergic asthma - AA). They are extremely common, affecting up to 30% of the population, have an increasing prevalence trend,^1^, ^2^, ^3^ and hold substantial sanitary, social, and economic implications.^4^ Apart from serious and potentially fatal complications (eg, severe asthma exacerbation), uncontrolled respiratory allergies negatively impact the quality of life (QoL) of affected patients and account for a remarkable healthcare expenditure.^5^, ^6^, ^7^, ^8^ Even data from non-life-threatening conditions such as AR are worrisome: there is a strong link between inadequately controlled AR and reduced QoL and work/school performances.^9^, ^10^, ^11^ Moreover, although the costs associated with AR are lower than those of asthma, AR is far more common. Thus, the associated expenditure becomes particularly relevant on a population basis.^8^ For example, studies indicated that total healthcare costs associated with AR in 1996 in the United States ranged between $ 1.9 and 3.4 billion and more recently appeared to rise up to $ 20.9 billion.^4^^,^^12^^,^^13^ Studies in European countries suggested an expenditure per AR patient that ranged between € 159 to 544 per patient each year, higher in patients with comorbid asthma.^14^, ^15^, ^16^

Standard pharmacologic therapy, together with allergen avoidance, are fundamental for the management of airborne allergies. Nevertheless, these measures do not modify the natural disease history of allergies, and many patients fail to obtain disease control with available therapies.^4^^,^^7^ Allergen immunotherapy (AIT) stands out as the only therapeutic approach that provides long-term tolerance to allergens and is based on the controlled administration of allergen extracts derived from the allergens responsible for patients’ clinical manifestations.^17^ AIT reprograms the immune system to develop tolerance towards the allergen, eg, by altering lymphocyte responses, adjusting the activation threshold of effector cells, and promoting the activity of regulatory cells.^17^ This approach is unique in its ability to change the natural progression of allergies, offering long-lasting relief for conditions like AR and AA.^18^^,^^19^

The 2 most common modalities of administration of AIT are subcutaneous (SCIT) and sublingual (SLIT). SCIT has standardized regimens and protocols due to the fact that it has been the most widely used immunotherapy approach for more than a century.^20^ In the former 3 decades, SLIT has been accepted as a viable alternative to SCIT.^21^ SLIT is affected by numerous variables due to possible different timings and doses. In particular, the maintenance dose and the time interval between each maintenance dose (such as daily, on alternate days, or twice weekly) strictly depend on the standardization method, which varies from 1 manufacturer to another.^20^^,^^21^ Both routes of administration carry benefits and drawbacks, and the choice between these 2 therapies depends on various factors involving the doctor and the patient. SCIT is associated with a higher rate of adverse effects, of which the vast majority are local and mild, but very rarely can result in life-threatening systemic allergic reactions;^22^ on the other hand, SLIT has a better safety profile but is associated with a lower adherence, which is a critical aspect for AIT efficacy.^23^^,^^24^

It is important to note that the evaluation of AIT efficacy can be challenging for 2 main reasons: 1) the wide variety of AIT products composition (efficacy has to be demonstrated for every product inside the different compositions rather than for a class);^25^ and 2) AIT studies are seldom comparable because of the diversity of allergen extracts, doses, and dosing regimens. Also, most studies differ in designs, inclusion criteria and outcome assessments.

Nevertheless, numerous studies demonstrated the efficacy of AIT for several allergens in AR and allergic asthma.^26^, ^27^, ^28^, ^29^, ^30^, ^31^ Besides, there is evidence that AIT also plays a preventive role, as it seems that in individuals treated with AIT for allergic rhinitis there is a lower future risk of developing AA.^32^^,^^33^ AIT also decreases the rate of onset of novel sensitizations, and polysensitization is linked to a higher degree of severity of respiratory allergies.^34^, ^35^, ^36^

Despite the number of studies providing evidence for AIT efficacy, this treatment continues underused.^37^ The reasons behind this might be multiple, including costs, a high heterogeneity among national guidelines worldwide, misinformation (eg, the false belief that AIT is unsafe), skepticism, information delivered by healthcare professionals and the severity of the underlying disease.^37^^,^^38^ However, there needs to be studies explicitly designed to assess the reasons behind this critical aspect. Understanding AIT utilization patterns might disclose information about why it is underused, thus providing valuable insights on how to broaden the positive impact it can have on the population. From an economic standpoint, AIT reduces on-demand drug consumption and the number of medical visits, bringing about cost advantages.^39^ Pharmacoeconomic studies are important as they allow decision-makers to decide where to allocate public funds better. Despite a lot of evidence supporting AIT cost-effectiveness, this treatment is reimbursed only in 56% of European countries (with full reimbursement in only 32%).^40^ AIT adherence is a critical aspect of AIT efficacy, and there is evidence that economic access is highly relevant in this context.^40^

The vast majority of Italian citizens do not have access to AIT reimbursement and there are only 3 AIT products registered as drugs: 2 for grass allergy (the health-care system can reimburse that) and 1 for house dust mites (which is reimbursed only in few regions). All other AIT products are classified as “named patient products”, and for them, AIT reimbursement access in Italy varies depending on the patient's region of residence, with total reimbursement in Lombardy and Apulia (the latter only for low-income individuals), partial reimbursement in Piedmont, and the remaining regions place the entire AIT financial burden on taxpayers. The direct result of this inequality is disproportionate access to this therapeutic intervention for Italian citizens.

This study aimed to analyze the trends in the use of AIT, particularly about 3 specific factors: the cost of treatment, patient health literacy concerning AIT, and the impact of allergic disease on patients in terms of symptoms as well as days lost for allergic diseases. Also, we aimed to assess if reimbursement policies for AIT in various regions of Italy could influence patients' decisions regarding whether to undergo the therapy and may also impact premature AIT discontinuation.

## Methods

This study has been conducted in collaboration with the national patient association *Respiriamo Insieme-APS*. Established in 2014, this association is registered in the *Registro Unico Nazionale del Terzo Settore* and has approximately 1900 members. They include patients affected by respiratory disorders, caregivers, families, as well as experts (such as allergists, anthropologists, pediatricians, pulmonologists, and psychologists) who form the scientific committee.

An online survey was created utilizing the modular Google® data collection platform (Google®-Google Forms®). The questionnaire aimed to assess the trends in the use of AIT in Italy, patient health literacy on AIT, and the impact of respiratory allergies and AIT on patients' lives. The questionnaire contained a first set of general demographic questions, followed by questions on patients' allergic condition and specific questions on patients' knowledge of, and experience with, AIT for respiratory allergy. In order to make the questions actually easy to understand and answer for patients, a preliminary version of it was initially submitted to and reviewed by 15 patients selected from the association, and improvements were made according to their feedback. The final version of the questionnaire was approved by the Ethics Committee “Campania Nord” (registry CECN/2098, 26-apr-2023) and can be found in Appendix A. Informed consent was obtained from all participants and children's’ parents included in the study. The collection of anonymous personal data was regulated by the personal data protection law (No. 67/98 of Oct-26). The questionnaire was submitted to the members of the association *Respiriamo Insieme-APS* during the period between May and October 2023, ensuring at least 1 response from each Italian region.

The collected data were analyzed using IBM SPSS® software version 23.0 (SPSS, Chicago, IL, USA). Categorical data were compared using contingency tables, and statistical testing was performed with the Pearson χ2 (Chi-square) test and Fisher exact test to adapt the statistical analysis to the sample size. Results were considered statistically significant if the p-values were equal to or less than 0.05.

## Results

**Fig. 1:**
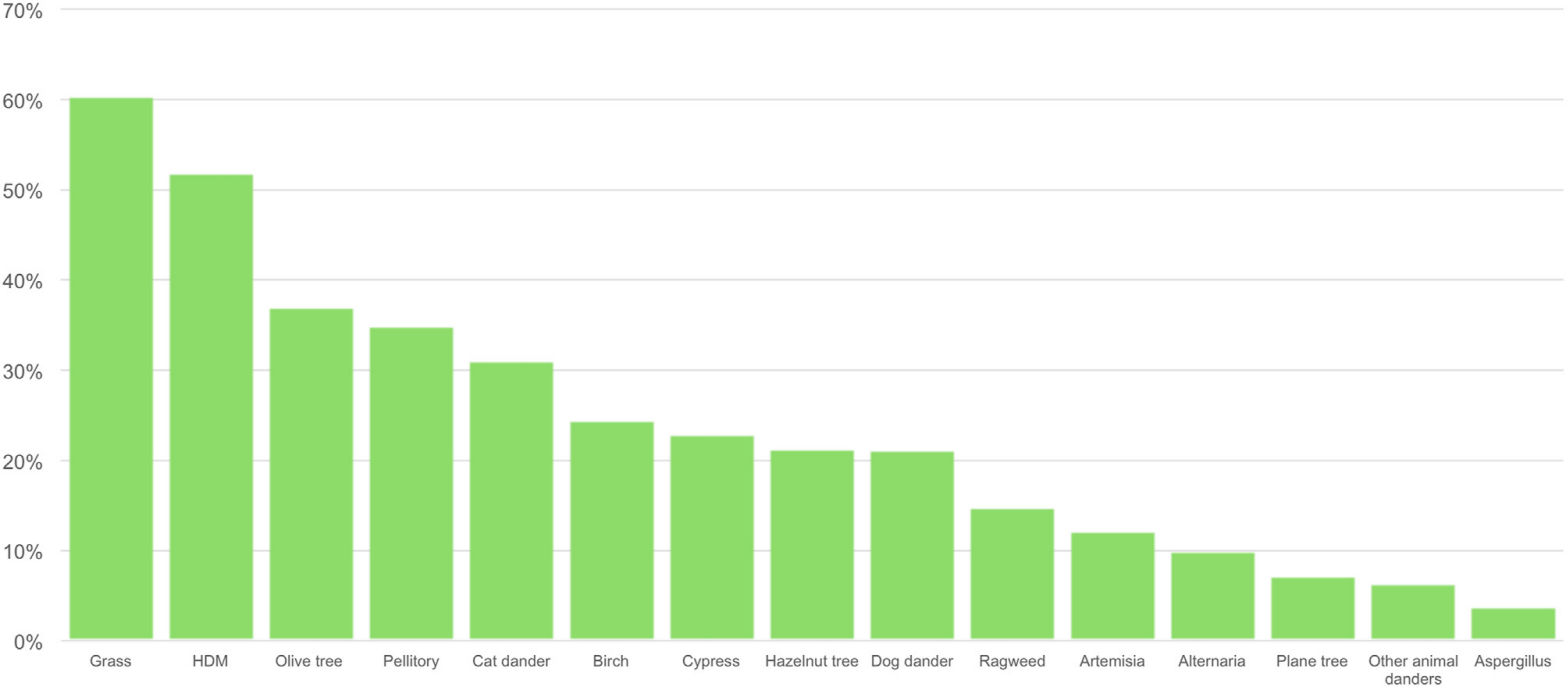
Distribution of patients' sensitizations. HDM: House Dust Mites

944 patients completed the questionnaire, of which 655 (69.4%) were females. The mean age was 35 (SD ± 16.8), and 177 (18.7%) were children (age <14 years) (in the latter case, questionnaires were filled by parents). Regarding the geographic distribution, 695 (73.6%) lived in a city, while the remaining 249 (26.4%) came from rural areas. There was at least 1 response from each Italian region, with Lombardy having the highest number of responses (n = 170, 18%) and Molise having the lowest (n = 3, 0.3%). The geographical distribution of responses according to the region of origin is reported in Supplemental Figure 1. Most patients reported to be affected by allergic rhinitis (n = 766, 81.1%), while others reported allergic asthma (n = 429, 45.4%), and/or allergic conjunctivitis (n = 389, 41.2%), with many patients presenting a combination of these diseases. The majority (n = 518, 54.9%) had a positive family history for allergic rhinitis (in first-degree family members). The 2 most frequently reported sensitizations were to grass pollen (n = 570, 60.4%) and house dust mites (HDM) (n = 490, 51.9%). The distribution of patients’ sensitizations is illustrated in Fig. 1.

**Fig. 2:**
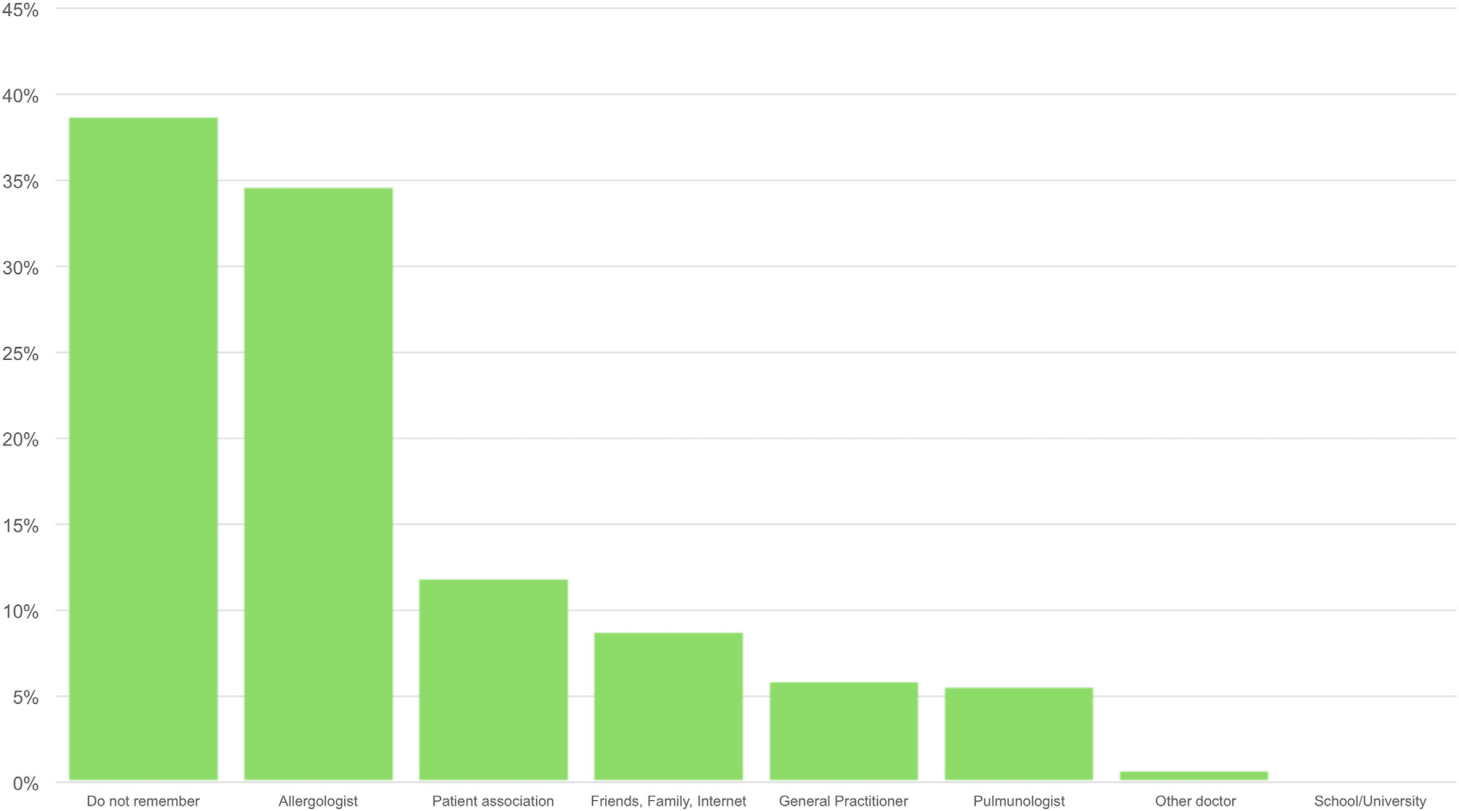
How patients learned about allergen immunotherapy existence

**Fig. 3:**
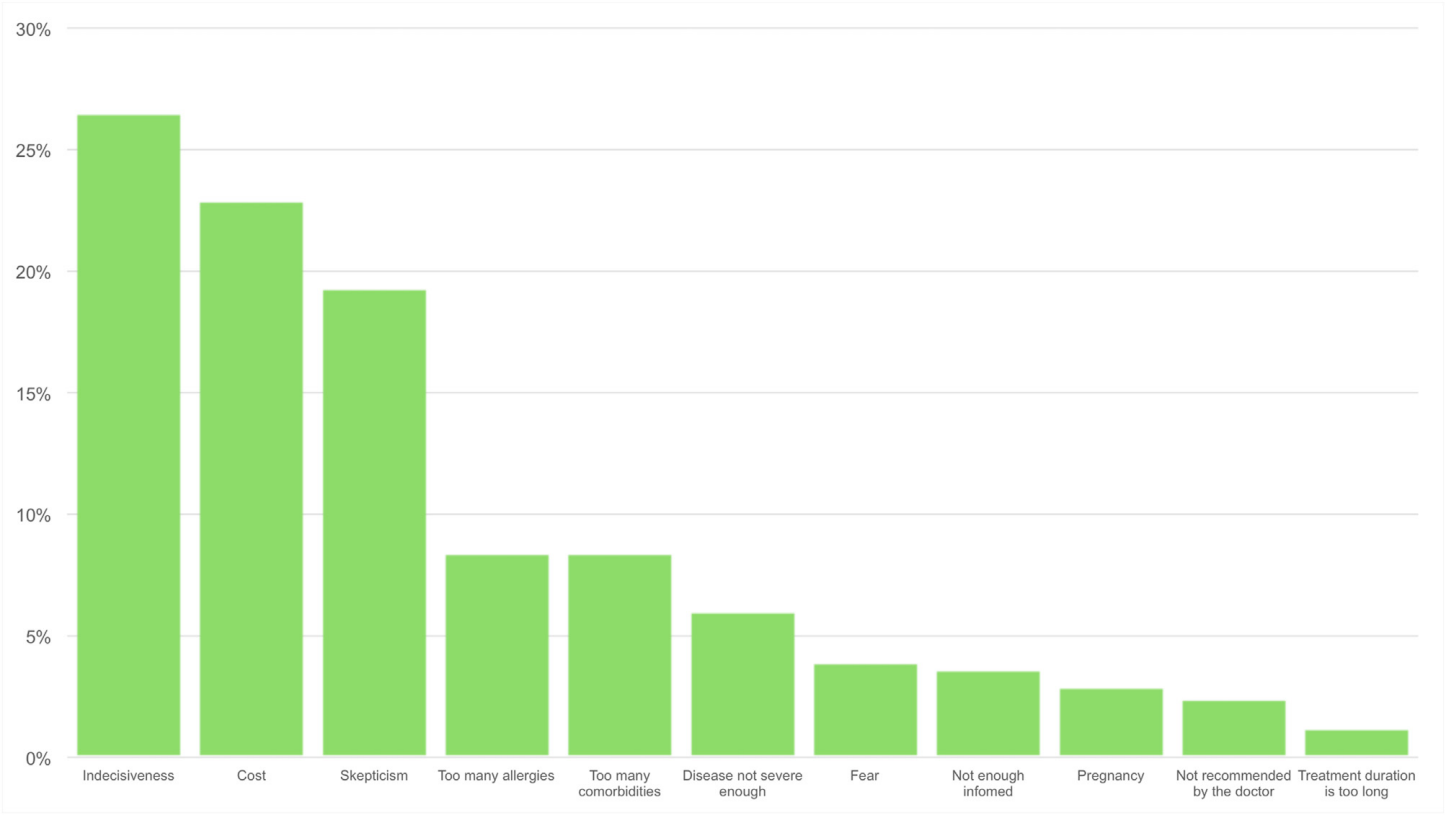
Reasons behind allergen immunotherapy refusal

Concerning the dedicated section on AIT, 508 patients (53.8%) knew about AIT existence with a significant difference between the knowledge of parents of children (n = 79, 44.6% of all children) and adult patients (n = 429, 55.9% of all adults), p = 0.007. Three-hundred and sixty-six patients (38.8%) did not remember how they learned about AIT existence, with the second largest group crediting their allergologist (n = 327, 34.6%); parents of allergic children received more frequently information about AIT by patients’ associations compared to adult patients (39 out of 177 children, 22.0% vs 73 out of 767 adult patients, p < 0.001). The distribution of answers on how patients got acquainted with AIT are shown in Fig. 2. Out of 858 patients suffering from allergic asthma and/or allergic rhinitis (the main indications for AIT for respiratory allergies in Italy), AIT was proposed to 284 (33.1%), with a significantly lower proportion for children (25.0%) compared to adult patients (35.0%), p = 0.01. Within the ones to whom AIT was proposed, 83 (29.2%) refused it. AIT was most frequently refused due to reasons referrable to indecisiveness (n = 22, 26.5%), costs (n = 19, 22.9%), and skepticism (n = 16, 19.3%). The distribution of the reasons behind AIT refusal are illustrated in Fig. 3.

**Fig. 4:**
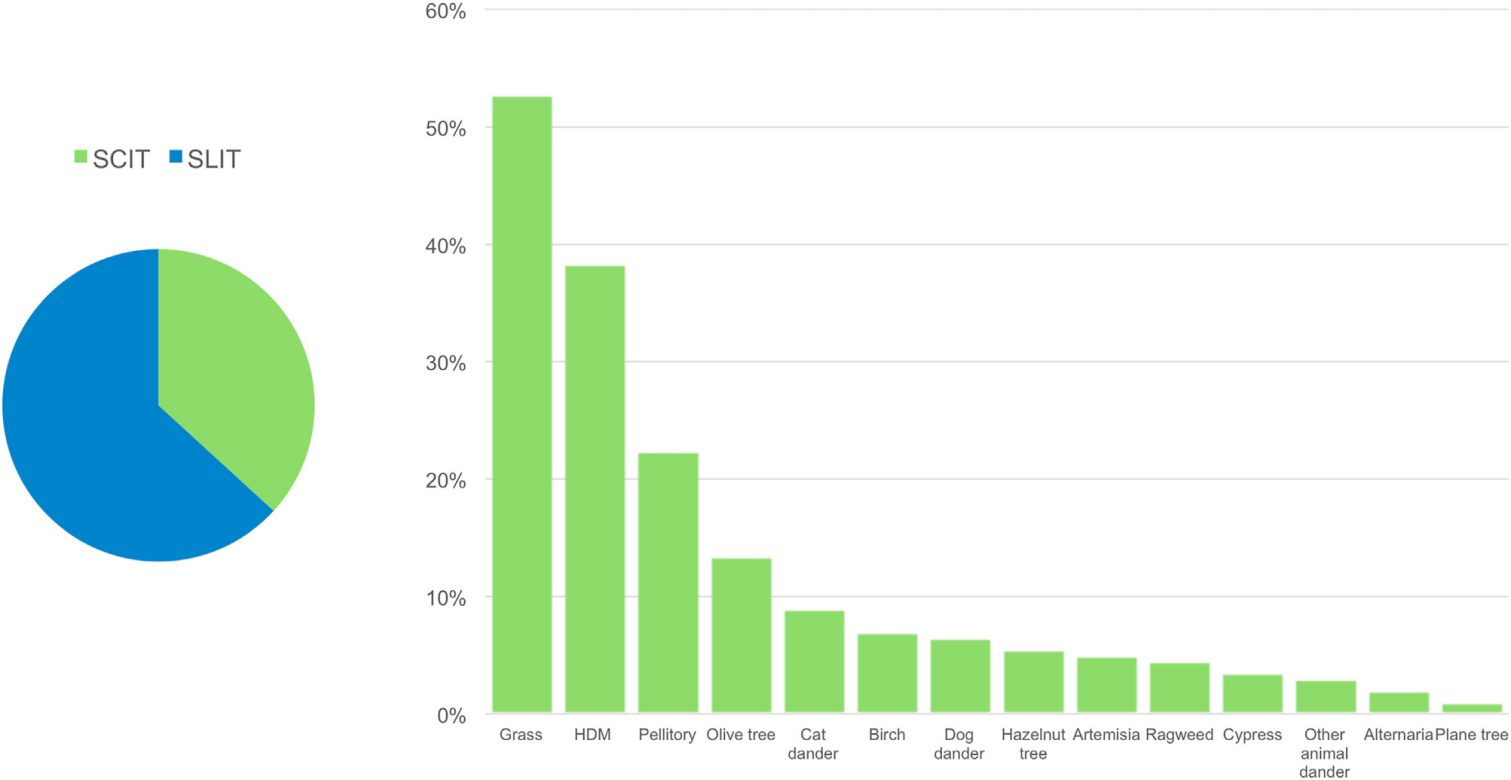
Allergens for which allergen immunotherapy was started (right panel) and administration routes (left panel)

Two hundred and one patients started AIT (70.8% to whom AIT was proposed), of which 127 (63.2%) underwent SLIT, while the reminder SCIT. The 2 most common allergens for which AIT was started were grass pollen (n = 106, 52.7%) and HDM (n = 77, 38.3%). The distribution of allergens for which AIT was prescribed is shown in Fig. 4.

**Fig. 5:**
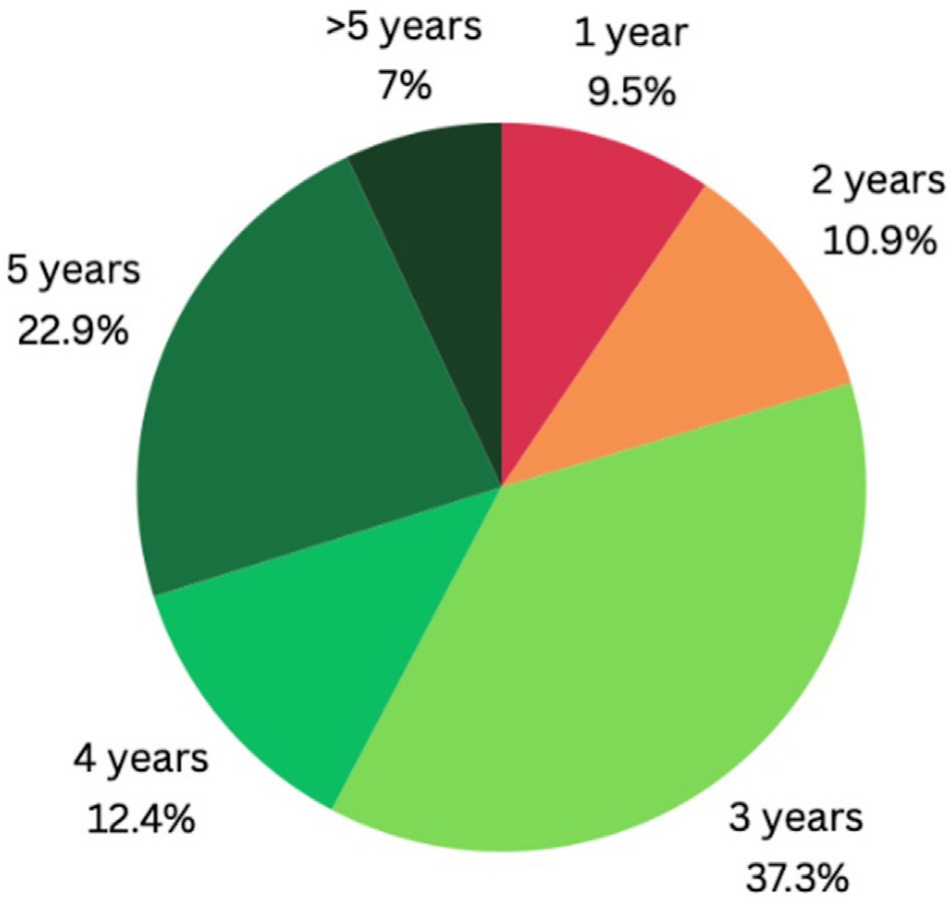
Duration of allergen immunotherapy prescription

There was a high variability in the duration of AIT prescription, with 9.5% of patients receiving indications to continue AIT for 1 year, 10.9% for 2 years, 37.3% for 3 years, 12.4% for 4 years, and 22.9% for 5 years. These data are depicted in Fig. 5.

**Fig. 6:**
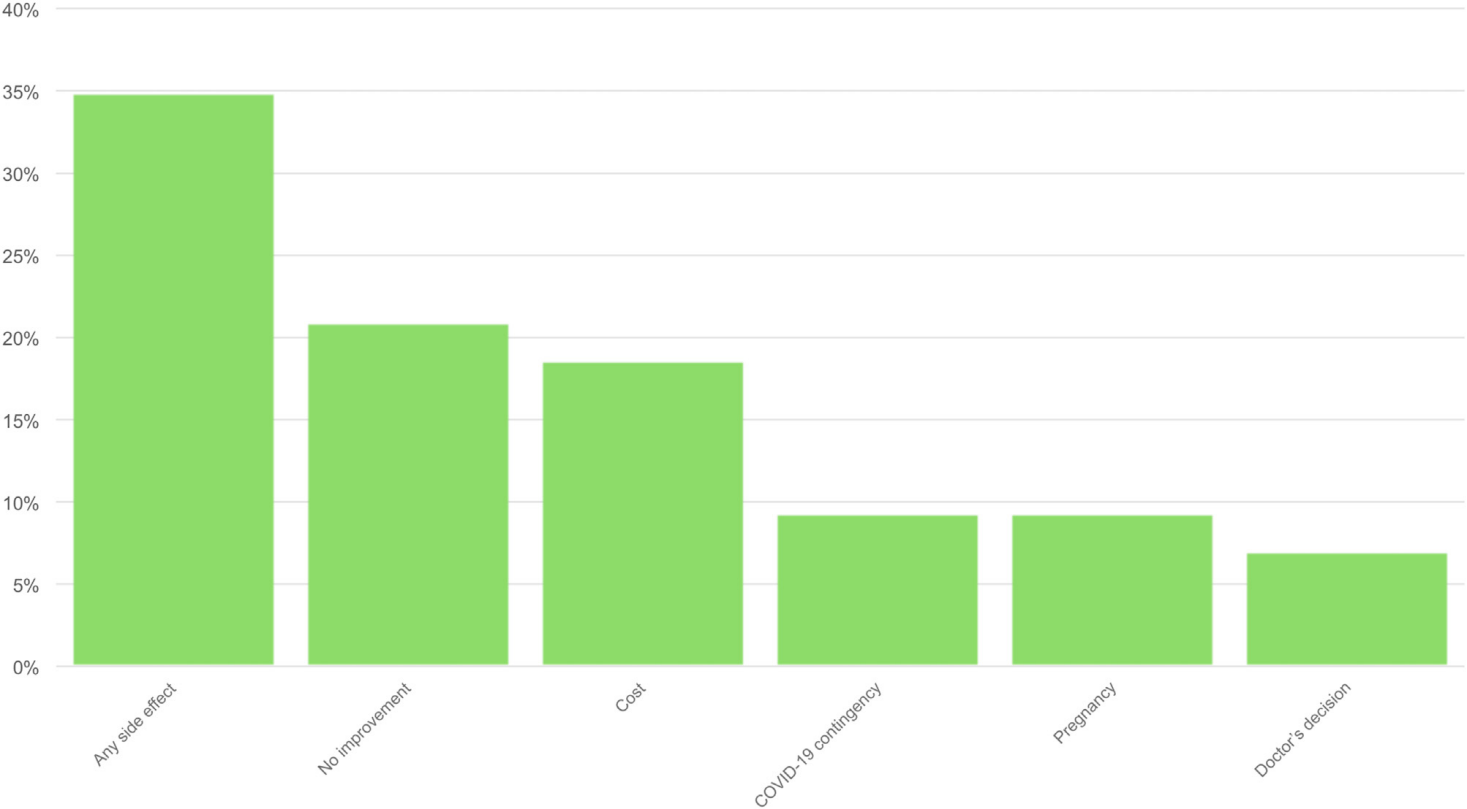
Reasons behind allergen immunotherapy discontinuation

Among those who started AIT, 43 (21.4%) discontinued the treatment beforehand with respect to the proposed duration, with a mean duration of AIT before interruption of 18.3±16.5 months. All patients who discontinued AIT were adults. The most frequent reasons for AIT discontinuation were adverse effects (n = 15, 34.9%), perception of no clinical improvement (n = 9, 20.9%) and costs (n = 8, 18.6%). Fig. 6 shows the distribution of the reasons behind AIT discontinuation. No significant difference was found in the discontinuation rate and in any of the reported side effects comparing SCIT and SLIT. Moreover both number and type of allergens to which patients were sensitized did not significantly differ in patients who discontinued AIT earlier (before 3 years) compared to those with a treatment duration lasting at least 3 years.

**Fig. 7:**
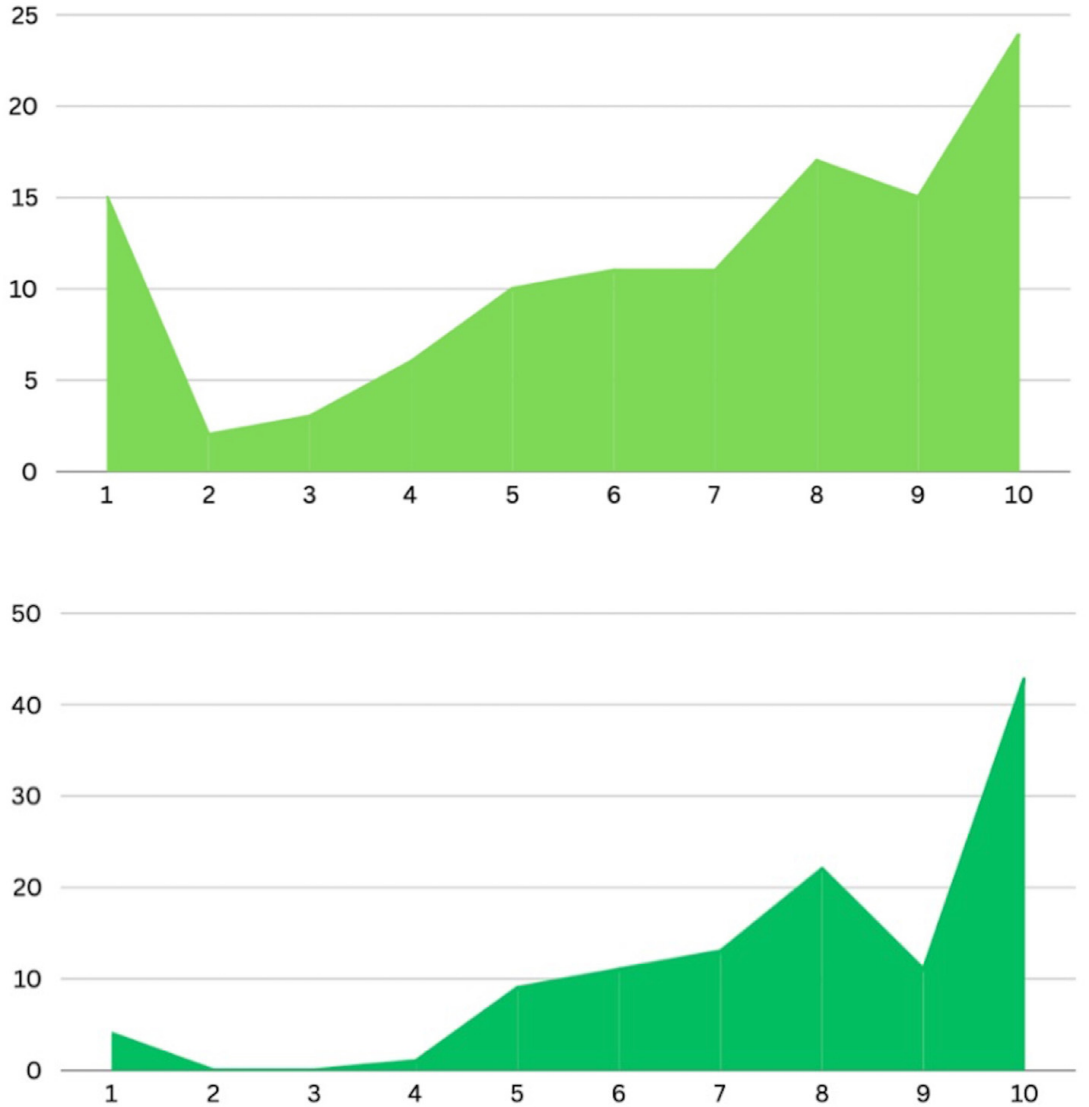
Perceived efficacy (upper panel) and safety (lower panel) of allergen immunotherapy in patients who completed it

**Fig. 8:**
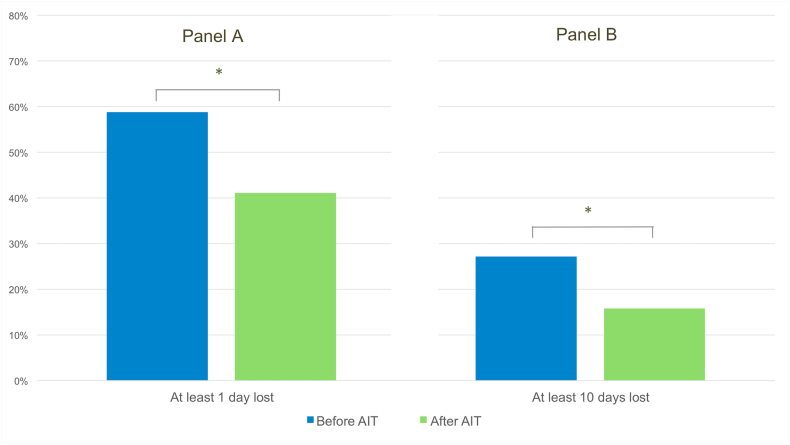
Workdays/schooldays lost before and after allergen immunotherapy

The final part of our analysis was on patients who completed AIT (n = 114), of which 36 (31.6%) believed the therapy to be of little use (≤5 on a scale of 1–10), while 78 (68.4%) believed it to be effective (>5), with the most chosen rating being 10 (by 24 patients) (Fig. 7, upper panel). As shown in Fig. 7, lower panel, 14 patients (12.3%) believed the therapy to be somewhat unsafe (≤5 on a scale of 1–10), while 86 (75.4%) believed it to be safe (>5), with the most chosen rating being 10 (by 43 patients). Lastly, a significant reduction in the percentage of patients reporting work or school days lost due to allergic rhinitis and/or allergic asthma before and after the administration of AIT was observed: patients who lost at least 1 work/school day in the previous year passed from 58.8% to 41.2% (p = 0.008) and those who lost at least 10 work/school days passed from 27.2% to 15.8% (p = 0.036) (Fig. 8). No significant difference was found in terms of reported effectiveness, safety and reduction in work or school days, comparing patients treated with SLIT or SCIT.

## Discussion

This real-world study shed light on the trends in the use of AIT, patients’ knowledge of it, their attitudes to initiating, continuing or discontinuing AIT, and their feelings about the impact of AIT on their lives. Moreover, to the best of our knowledge, this is 1 of the few reports in which the relationship between the accessibility of AIT and the heterogeneity of costs among Italian regions was assessed. The only other Italian study that explored the same aspect is now 14 years old and no longer fully reflects the AIT reimbursement situation in Italy.^41^ However, that study also highlighted lower adherence to therapy starting from the second year of treatment in patients living in regions where AIT was not reimbursed.

First of all, it has emerged that there is still much ground to cover to properly inform patients affected by airborne allergies about the existence, efficacy, and safety of AIT, particularly in pediatric setting where the role of patients’ associations make up for the lack of information provided by healthcare professionals who are also likely responsible for the lower proportion of patients to whom AIT is offered as a therapeutic option. Moreover, despite more than half of the respondents being aware of AIT, over one-third of patients needed to be made aware of how they came to know about it. These data hold particular significance, particularly in light of the ongoing historical period characterized by a surge in misinformation on health topics following the recent global medical emergency, highlighting the potential for misinformation when the source of information is unknown.^42^ The lack of knowledge of AIT is also reflected by the reasons patients decided not to undergo AIT when it was proposed by a physician, frequently attributable to skepticism (about 20%) and indecisiveness (more than 25%). These data corroborate with existing findings in the scientific literature, highlighting the prevalence of misinformation in the general population about allergies and AIT, a trend that the scientific community has been actively trying to counteract through the publication of numerous studies on the efficacy and safety of AIT.^43^^,^^44^ The disparity between misinformed and well-informed patients is evident when considering those who completed the treatment. Patients who completed their AIT treatment rated it both useful and safe. Particularly noteworthy is that the most common rating on a scale from 1 to 10 for both therapy safety and usefulness was 10. Therefore, while many patients decided against AIT due to skepticism or indecision, most patients who were adequately informed and thus undertook and completed the treatment were fully satisfied with both its efficacy and safety. Another finding emphasizing misinformation on AIT indirectly concerns physicians. Among patients who underwent AIT, there was no uniformity in the duration of therapy, indicating that physicians themselves needed to be properly informed about the necessary timing for AIT to be effective. Specifically, only 37.3% underwent AIT for 3 years, the recommended duration for the efficacy of this treatment,^45^ and 20.4% of patients were recommended a therapy duration below 3 years, which is not sufficient to obtain the expected results.^46^ Further studies should be conducted to understand the etiology of this misinformation better, investigating both the medical and general populations to comprehend the roots of the problem and address them effectively. In practice, part of the issue could be resolved by directly addressing doctors through specific questionnaires to assess their level of education regarding AIT and provide training and educational materials to make them better health advocates. Also, patient associations represent a precious ally for healthcare professionals in delivering high-quality information to patients, particularly for pediatric patients and their families. Our results align with those of a recent study conducted in China, which investigated attitudes towards AIT among patients with allergic rhinitis.^47^ The study revealed insufficient knowledge, unfavorable attitudes, and suboptimal practices regarding AIT, highlighting the need for targeted educational interventions.

Regarding assessing the impact of airborne allergies and AIT on patients' lives, our study confirms that AIT can alleviate disease burden in real-world practice. The majority of responders that completed AIT found it safe and effective. Importantly, we analyzed the number of work/school days lost due to AR and/or AA before and after the administration of AIT, demonstrating that AIT administration significantly reduces the number of days lost due to allergic conditions. Indeed, a significant reduction was observed when considering both patients who lost at least 1 day of work/school and those who lost at least 10 days before and after AIT administration. The results obtained are also consistent with existing literature,^48^ pointing out that in the case of the Italian population, AIT reduces sick days and improves patients' lives. Altogether, these findings suggest a benefit on patients’ QoL, spanning from improving clinical manifestations to reducing work absenteeism.

Another aspect investigated in this study is the issue of refundability and the impact that AIT-related costs can have on its use trends. Notably, cost was a frequently reported reason for AIT refusal as well as for early discontinuation. The lack national laws regulating the refundability of AIT results in interregional cost differences, and we hypothesized that this could significantly influence attitudes and choices regarding AIT across distinct Italian regions.

The main limitations of this study are the sample size and the need for supplementary data from patients, which could have provided a better insight into the investigated causality. For example, data on income, socio-economic status, family size, and other diseases requiring non-refundable treatment could have allowed us to draw additional conclusions as well as to reduce the risk of bias associated with these variables. Another bias in this study could arise from the fact that the association *Respiriamo Insieme-APS*, which administered the questionnaire to its members, is more active in certain regions and less active in others, leading to regional disparities in responses in the selected sample.

## Conclusions

This real-world study provided relevant insights into AIT usage, drawing directly from patients’ perspectives. Notably, many eligible patients opted not to start AIT, and many patients discontinued treatment beforehand. A high degree of heterogeneity in prescription patterns was also observed. Altogether, these issues seem to stem from considerable misinformation on AIT as well as the lack of refundability. The results of this study call for improvements in AIT awareness, information delivered about AIT efficacy and safety, standardization in AIT prescription patterns and economic accessibly.

## Abbreviations

AA: Allergic Asthma; AC: Allergic Conjunctivitis; AIT: Allergen Immunotherapy; AR: Allergic Rhinitis; HDM: House Dust Mites; QoL: Quality of Life; SCIT: Subcutaneous Immunotherapy; SLIT: Sublingual Immunotherapy.

## Funding

No funds were received for this study.

## Availability of data and materials

Raw data will be available upon requests to the Authors.

## Authors’ consent for publication

All the Authors read and approved the final version of the manuscript, and gave their consent for publication.

## Ethical approval

The study protocol was approved by the Ethics Committee “Campania Nord” (registry CECN/2098, 26-apr-2023).

## Declaration of competing interest

Giovanni Paoletti reports fees for speaker activities and/or advisory boards participation from Lofarma, GSK, and AstraZeneca, outside the submitted work.

Mattia Giovanini reports personal fees from Sanofi, outside the submitted work.

Giorgio Walter Canonica reports research or clinical trials grants paid to his Institution from Menarini, AstraZeneca,GSK, Sanofi Genzyme and fees for lectures or advisory board participation from Menarini, AstraZeneca, CellTrion, Chiesi, Faes Farma, Firma, Genentech, Guidotti-Malesci, GSK, HAL Allergy, Innovacaremd, Novartis, OM-Pharma, Red Maple, Sanofi-Aventis, Sanofi-Genzyme, Stallergenes-Greer and Uriach Pharma, outside the submitted work.

Enrico Heffler reports fees for speaker activities and/or advisory boards participation from Sanofi, Regeneron, GSK, Novartis, AstraZeneca, Stallergenes-Greer, Chiesi, Almirall, Bosch, Lofarma, outside the sumitted work.

Emanuele Nappi, Maria Chiara Bragato, Paola Valli, Domenico Gargano, Luca Pecoraro, Deborah Diso and Simona Barbaglia report no conflicts of interest.

## Appendix A

### Survey – English Version

*The original version of the survey in Italian is below the following translated version*.

**I am filling out the questionnaire**

• For myself
• Expressing clinical data and my impressions of my son/daughter (in this case, all subsequent questions refer to the minor)
• Interviewing another adult person (in this case, all subsequent questions refer to the other adult person)

**Biological Sex**

• Male
• Female

**Date of Birth**

• Specify the date

**I live in a …**

• Urban area
• Rural area

**Specify the region where you live**

• Abruzzo
• Basilicata
• Calabria
• Campania
• Emilia Romagna
• Friuli Venezia Giulia
• Lazio
• Liguria
• Lombardy
• Marche
• Molise
• Piemonte
• Apulia
• Sardinia
• Sicilia
• Toscana
• Trentino Alto Adige
• Umbria
• Val d’Aosta
• Veneto

**I suffer from**:

• Allergic rhinitis
• Allergic conjunctivitis
• Allergic asthma
• Non-allergic asthma
• Atopic dermatitis
• Chronic idiopathic urticaria
• Food allergy
• Insect sting allergy (Hymenoptera)
• Eosinophilic esophagitis
• Nasal polyposis
• Drug allergy (if documented by an allergist)
• None of these conditions

**Family history (understood as Mother and/or Father and/or siblings and/or children) affected by:**

Grid with checkboxes

Rows:

• Father
• Mother
• Brother/Sister
• Son/Daughter

**Columns**:

• None of these conditions
• Allergic rhinitis
• Allergic conjunctivitis
• Allergic asthma
• Non-allergic asthma
• Atopic dermatitis
• Chronic idiopathic urticaria
• Food allergy
• Insect sting allergy (Hymenoptera)
• Eosinophilic esophagitis
• Nasal polyposis
• Drug allergy (if documented by an allergist)

**I am sensitized to (positive prick test or positive blood test even without symptoms to)**:

• No inhalant allergen
• Composites
• Grasses
• Cypress
• Hazel
• Pellitory
• Olive
• Ragweed
• Alternaria
• Aspergillus
• Plane tree
• Birch
• Dust mites
• Cat dander
• Dog dander
• Other animal dander (excluding cat and dog)

**If affected: Year of onset of allergic asthma**

• Specify the date

**If affected: Who primarily follows you for allergic asthma problems**

• I am not affected by allergic asthma
• General Practitioner
• Pediatrician
• Allergist
• Pulmonologist
• Other … (specify)

**If affected: Who primarily follows you for allergic rhinitis problems**

• I am not affected by allergic rhinitis
• General Practitioner
• Pediatrician
• Allergist
• Pulmonologist
• Other … (specify)

**Do you know what specific inhalant immunotherapy is (allergy vaccine/desensitizing therapy)?**

• Yes
• No

**If you know what allergen immunotherapy for airborne allergens is (allergy vaccine/desensitizing therapy), who informed you?**

• I don't know what desensitizing therapy/allergy vaccine/allergy immunotherapy is
• Family doctor
• Pulmonologist
• Allergist
• A patient association
• Internet
• Other … (specify)

**Has allergen immunotherapy for airborne allergens (allergy vaccine/desensitizing therapy) ever been proposed to you by the doctor who follows you?**

• Yes for allergic asthma *→ If this answer is selected, the questionnaire continues with the following questions*
• Yes for allergic rhinitis → *If this answer is selected, the questionnaire continues with the following questions*
• Yes for both allergic asthma and allergic rhinitis → If this answer is selected, the questionnaire continues with the following questions
• No → *If this answer is selected, the questionnaire ends here*
• I don't have these conditions → *If this answer is selected, the questionnaire ends here*

**If it was proposed, did you undergo it?**

• Yes → *If this answer is selected, skip the next question and proceed to the following* questions No → *If this answer is selected, proceed to the next question*

### **If allergen immunotherapy was proposed to you and you did not start it, what was the reason?**

• Cost
• I can't always take the treatments consistently, and I thought it wouldn't work
• I didn't believe it could help me
• Other … (specify)

### **In which year did you start allergen immunotherapy?**

• Specify the date

**In which region was it prescribed?**

• Abruzzo
• Basilicata
• Calabria
• Campania
• Emilia Romagna
• Friuli Venezia Giulia
• Lazio
• Liguria
• Lombardy
• Marche
• Molise
• Piedmont
• Apulia
• Sardinia
• Sicily
• Tuscany
• Trentino Alto Adige
• Umbria
• Val d’Aosta
• Veneto

**Did you pay for the therapy?**

• The therapy was fully paid for through the national or regional health system
• The therapy was partially paid for through the national or regional health system
• I paid for the therapy completely

**Was it prescribed as**.

• Sublingual therapy
• Injectable therapy

**For which allergen(s) was it prescribed?**

• Composites
• Grasses
• Cypress
• Hazel
• Pellitory
• Olive
• Ragweed
• Alternaria
• Aspergillus
• Plane tree
• Birch
• Dust mites
• Cat dander
• Dog dander
• Other animal dander (excluding cat and dog)

**For how many years were you advised to continue the therapy in total?**

• 1 year
• 2 years
• 3 years
• 4 years
• 5 years
• More than 5 years

**How many work or school days did you miss in the year before allergen immunotherapy due to rhinitis and/or asthma?**

• 0
• From 1 to 10 days
• From 11 to 30 days
• More than 31 days

**Did you stop allergen immunotherapy before the times indicated by the doctor who prescribed it to you?**

Yes → *If this answer is selected, proceed to the next question*.

No → *If this answer is selected, skip the next two questions and proceed to the following questions*.

**If you stopped it, after how many months (express in number of months, NOT in words)?**

• Specify the number of months

**If you stopped it, what was the reason?**

• Cost
• I can't always take the treatments consistently, and I thought it wouldn't work
• I didn't believe it could help me
• Other … (specify)

**Have you completed the years of allergen immunotherapy recommended by your doctor?**

• Yes
• No, it is currently ongoing

**If you completed it, how many work or school days did you miss in the year following the end of allergen immunotherapy due to rhinitis or asthma?**

• I did not complete the therapy
• 0
• From 1 to 10 days
• From 11 to 30 days
• More than 31 days

**Do you think the therapy was useful? Give a score from 1 to 10**

Linear scale from 1 to 10.

• 1: No therapeutic result
• 10: Excellent therapeutic result

**Do you think the therapy was safe? Give a score from 1 to 10**

Linear scale from 1 to 10.

• 1: Not safe at all
• 10: Completely safe

## Survey – Original Version in Italian

**Sto compilando il questionario**

• Per me stesso
• Esprimendo i dati clinici e le mie impressioni su mia figlio/mia figlia (in tal caso tutte le domande che seguiranno sono da riferirsi al minore)
• Intervistando un' altra persona adulta (in tal caso tutte le domande che seguiranno sono da riferirsi all'altra persona adulta)

**Sesso Biologico**

• Maschio
• Femmina

**Data di nascita**

• Specificare la data

**Abito in …**

• Ambiente cittadino
• Ambiente rurale

**Regione dove abiti**

• Abruzzo
• Basilicata
• Calabria
• Campania
• Emilia Romagna
• Friuli Venezia Giulia
• Lazio
• Liguria
• Lombardia
• Marche
• Molise
• Piemonte
• Puglia
• Sardegna
• Sicilia
• Toscana
• Trentino Alto Adige
• Umbria
• Val d’Aosta
• Veneto

**Sono affetto da**:

• Rinite allergica
• Congiuntivite allergica
• Asma allergico
• Asma non allergico
• Dermatite Atopica
• Orticaria cronica idiopatica
• Allergia alimentare
• Allergia verso gli imenotteri (insetti pungitori)
• Esofagite eosinofila
• Poliposi nasale
• Allergia a farmaci (se documentata da un allergologo)
• Nessuna di queste patologie

**Famigliarità (intendersi come Madre e/o Padre e/o fratelli e sorelle e/o figli/e) affetti da:**

**Righe:**

Griglia con caselle di controllo

• Padre
• Madre
• Fratello/Sorella
• Figlio/Figlia
• Colonne:
• Nessuna di queste patologie
• Rinite allergica
• Congiuntivite allergica
• Asma allergico
• Asma non allergico
• Dermatite Atopica
• Orticaria cronica idiopatica
• Allergia alimentare
• Allergia verso gli imenotteri (insetti pungitori)
• Esofagite eosinofila
• Poliposi nasale
• Allergia a farmaci (se documentata da un allergologo)

**Sono sensibilizzato verso (prick test positivo o test ematico positivo anche senza sintomi verso)**

• Nessun allergene inalante
• Composite
• Graminacee
• Cipresso
• Nocciolo
• Parietaria
• Olivo
• Ambrosia
• Alternaria
• Aspergillo
• Platano
• Betulla
• Acari
• Epitelio di gatto
• Epitelio di cane
• Epitelio di altri animali (escluso gatto e cane)

**Se affetto: Anno insorgenza dell'Asma allergico**

• Specificare la data

**Se affetto: Chi ti segue principalmente per la problematica dell’Asma allergica**

• Non sono affetto da Asma allergico
• Medico di Medicina Generale
• Pediatra
• Allergologo
• Pneumologo
• Altro … (specificare)

**Se affetto: Chi ti segue principalmente per la problematica della Rinite allergica**

• Non sono affetto da Rinite allergica
• Medico di Medicina Generale
• Pediatra
• Allergologo
• Pneumologo
• Altro … (specificare)

**Sai cosa sia l'immunoterapia specifica per inalanti (vaccino per le allergie/terapia desensibilizzante)?**

• Si
• No

**Se sai cosa sia l'immunoterapia specifica per inalanti (vaccino per le allergie/terapia desensibilizzante), chi ti ha informato?**

• Non so cosa sia la terapia desensibilizzante/vaccino per le allergie/immunoterapia per le allergie
• Il medico di famiglia
• Lo Pneumologo
• L'allergologo
• Un'associazione dei pazienti
• Internet
• Altro … (specificare)

**Ti è mai stata proposta immunoterapia specifica per inalanti (vaccino per le allergie/terapia desensibilizzante) dal medico che ti segue?**

• Si per l'asma allergica → Se viene selezionata questa risposta il questionario prosegue con le domande successive
• Si per la rinite allergica → *Se viene selezionata questa risposta il questionario prosegue con le domande successive*
• Si sia per l'asma allergico che per la rinite allergica → *Se viene selezionata questa risposta il questionario prosegue con le domande successive*
• No → *Se viene selezionata questa risposta il questionario termina qui*
• Non ho queste patologie → *Se viene selezionata questa risposta il questionario termina qui*

**Se ti è stata proposta la hai effettuata?**

• Si → *Se viene selezionata questa risposta si salta la prossima domanda e si passa alle domande successive*
• No → *Se viene selezionata questa risposta si procede con la prossima domanda*

**Se ti è stata proposta e non l'hai iniziata quale è stato il motivo?**

• Costo
• Non sempre riesco a prendere le terapie con costanza e ho pensato non avrebbe quindi funzionato
• Non credevo mi potesse aiutare
• Altro … (specificare)

**In quale anno hai iniziato la terapia desensibilizzante?**

• Specificare la data

**In quale regione ti è stata prescritta**

• Abruzzo
• Basilicata
• Calabria
• Campania
• Emilia Romagna
• Friuli Venezia Giulia
• Lazio
• Liguria
• Lombardia
• Marche
• Molise
• Piemonte
• Puglia
• Sardegna
• Sicilia
• Toscana
• Trentino Alto Adige
• Umbria
• Val d’Aosta
• Veneto

**Hai pagato la terapia**

• La terapia è stata pagata completamente attraverso il sistema sanitario nazionale o regionale
• La terapia è stata pagata in parte attraverso il sistema sanitario nazionale o regionale
• Ho pagato la terapia completamente

**Ti è stata prescritta**

• Terapia sublinguale
• Terapia iniettiva

**Per quale allergene/i ti è stata prescritta**:

• Composite
• Graminacee
• Cipresso
• Nocciolo
• Parietaria
• Olivo
• Ambrosia
• Alternaria
• Aspergillo
• Platano
• Betulla
• Acari
• Epitelio di gatto
• Epitelio di cane
• Epitelio di altri animali (escluso gatto e cane)

**Per quanti anni ti è stato consigliato di proseguire in totale la terapia**

• 1 anno
• 2 anni
• 3 anni
• 4 anni
• 5 anni
• oltre i 5 anni

**Quanti giorni lavorativi o scolastici hai perso nell'anno precedente dell'inizio dell'immunoterapia specifica (Vaccino per gli inalanti) per colpa della rinite e/o dell'asma?**

• 0
• Da 1 a 10 giorni
• Da 11 a 30 giorni
• oltre 31 giorni

**Hai interrotto l'immunoterapia prima delle tempistiche indicate dal medico che te la ha prescritta?**

• Si → *Se viene selezionata questa risposta si procede con la prossima domanda*
• No → *Se viene selezionata questa risposta si saltano le prossime due domande e si passa alle domande successive*

**Se la hai interrotta dopo quanti mesi (da esprimere in numero di mesi NON in lettere)?**

• Specificare il numero di mesi

**Se la hai interrotta quale è stato il motivo?**

• Costo
• Non sempre riesco a prendere le terapie con costanza e ho pensato non avrebbe quindi funzionato
• Non credevo mi potesse aiutare
• Altro … (specificare)

**Hai concluso gli anni di immunoterapia consigliata dal tuo medico**

• Si
• No è attualmente in corso

**Se la hai conclusa, quanti giorni lavorativi o scolastici hai perso nell'anno successivo al termine dell'immunoterapia specifica (Vaccino per gli inalanti) per colpa della rinite o dell'asma**

• Non ho concluso la terapia
• 0
• Da 1 a 10 giorni
• Da 11 a 30 giorni
• oltre i 31 giorni

**Reputi che la terapia sia stata utile? Dai un punteggio da 1 a 10**

**Scala lineare da 1 a 10**.

• 1: Nessun risultato terapeutico
• 10: Ottimo risultato terapeutico

**Reputi che la terapia sia stata sicura? Dai un punteggio da 1 a 10**

**Scala lineare da 1 a 10**.

• 1: Per nulla sicura
• 10: Del tutto sicura
