# Supplementary figures and images for: Allergen immunotherapy in Italy: How, when, and why—A real-world study conducted through a patient association
Source: World Allergy Organ J. 2024 Dec 24;18(1):101015. doi: 10.1016/j.waojou.2024.101015 (PMC11732536; doi:10.1016/j.waojou.2024.101015)

**Supplemental Figure 1** – Distribution of answers by Italian regions

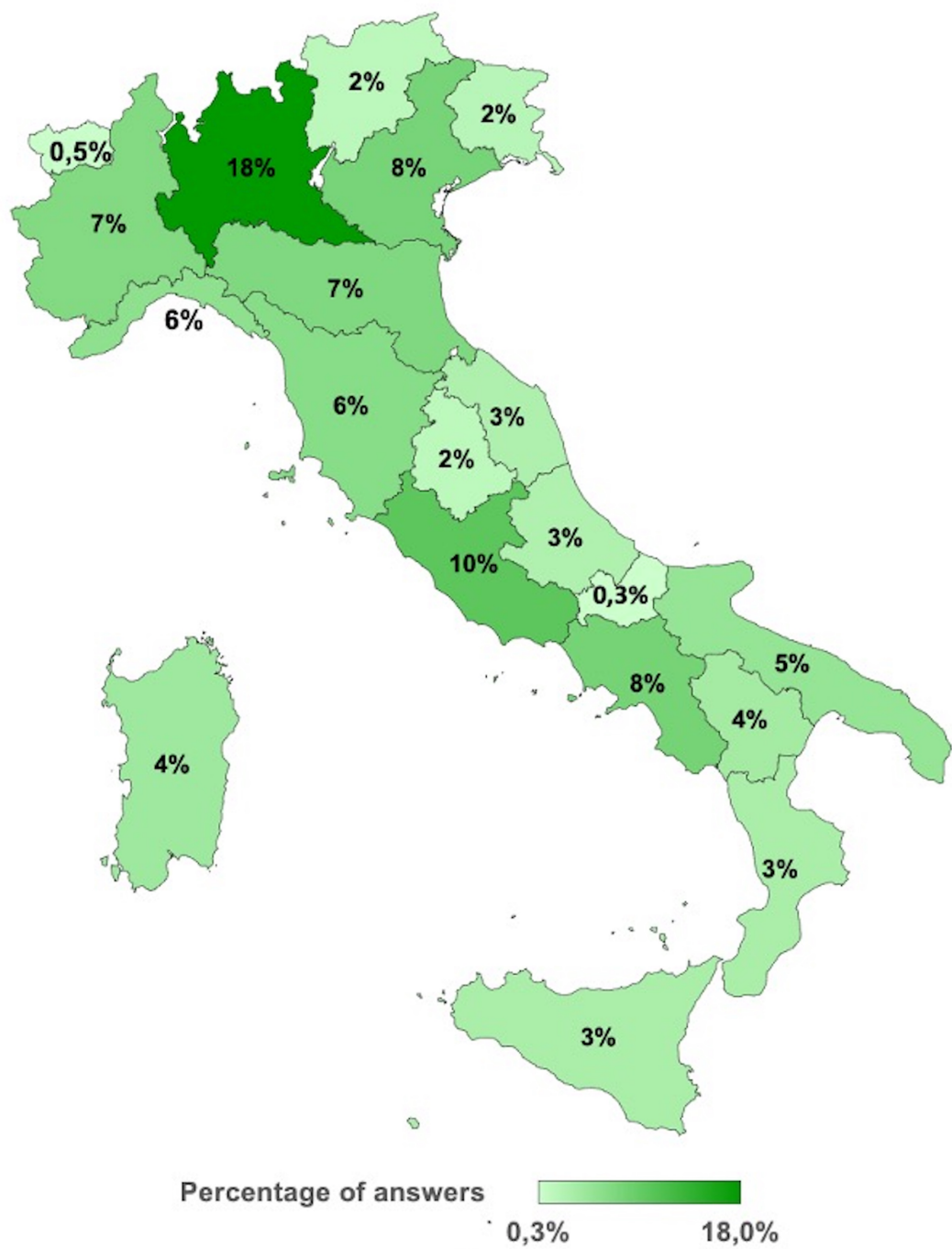

Supplement: Multimedia component 1 [file mmc1.pdf]
